# ‘We are always desperate and will try anything to conceive’: The convoluted and dynamic process of health seeking among women with infertility in the West Coast Region of The Gambia

**DOI:** 10.1371/journal.pone.0211634

**Published:** 2019-01-31

**Authors:** Susan Dierickx, Julie Balen, Chia Longman, Ladan Rahbari, Ed Clarke, Bintou Jarju, Gily Coene

**Affiliations:** 1 Centre of Expertise on Gender, Diversity and Intersectionality (RHEA), Vrije Universiteit Brussel, Brussel, Belgium; 2 Centre for Research on Culture and Gender, Ghent University, Ghent, Belgium; 3 School of Health and Related Research, The University of Sheffield, Sheffield, United Kingdom; 4 Vaccines and Immunity Theme, Medical Research Council Unit The Gambia at the London School of Hygiene and Tropical Medicine, Fajara, The Gambia; Lancaster University, UNITED KINGDOM

## Abstract

**Introduction:**

In many Sub-Saharan African countries, women with infertility search relentlessly for treatment. Guided by the Partners for Applied Social Sciences model for health seeking behaviour and access to care research, this study aims to understand the health seeking behaviour of women with infertility in the West Coast region of The Gambia and the influence of aetiological beliefs on health seeking paths.

**Methodology:**

A qualitative approach was used to generate both primary and secondary data for thematic analysis. The data collection methods included in-depth interviews (36), observations (18), informal conversations (42), group discussion (7) and made use of pile-sorting exercises. Sources of secondary data included government and non-governmental reports and media outputs.

**Results:**

The health seeking approaches of women living in both rural and urban areas were extremely complex and dynamic, with women reporting that they looked for biomedical treatment as well as seeking indigenous treatment provided by local healers, sacred places and *kanyaleng* groups. While treatment choice was related to the perceived aetiology of infertility, it was also strongly influenced by the perceived effectiveness of the treatment available and the duration of the fertility problems. Other relevant factors were the affordability, accessibility and availability of treatment and respondents’ family and social networks, whereby access to the biomedical health sector was strongly influenced by people’s socio-economic background.

**Conclusion:**

On the basis of this analysis and our wider research in the area, we see a need for health authorities to further invest in providing information and counselling on issues related to infertility prevention and treatment. The availability of locally applicable guidelines for the management of infertility for both men and women at all levels of the health system would facilitate such work. In addition, the public sphere should provide more space for alternative forms of social identity for both men and women.

## Introduction

Infertility is a global reproductive health problem affecting 48.5 million couples worldwide [1]. Especially in Sub-Saharan Africa (SSA) many people are confronted with primary infertility (1.9%) and there continues to be a high rate of secondary infertility (11.6%). However, there is no consistent clinical, epidemiological and demographical definition of infertility nor a common denominator to measure infertility [1–3]. Moreover, clinical, epidemiological and demographical conceptualizations of infertility, subfertility, miscarriage and stillbirth are not always congruent with people’s own understanding of these events [4–10]. Therefore, this study starts from women’s lived experiences and perceptions of infertility and includes women that consider themselves to have fertility problems, regardless of the duration of these problem or whether they relate to primary or secondary infertility.

Infertility has severe consequences for couples, especially for women living in SSA countries where female identity, social status and financial security often depend on the ability to produce offspring [2,5,11]. Women are frequently blamed for fertility problems and are confronted with general emotional distress, loss of marital stability, stigmatization and isolation at the community level [12,13]. As a result, infertility is much more than a medical condition and implies an additional heavy social burden for women to seek appropriate treatment. Access to biomedical health services directed at preventing and treating infertility remains very limited, or is non-existent, across SSA, although this is slowly changing in some countries [14,15]. Studies of health seeking behaviour and provider choice have problematized the decision of women with infertility to visit indigenous healers and seek spiritual treatment in order to be cured. This decision is often attributed to ‘traditional’ beliefs in witchcraft, spirits or God [2,16].

This research was conducted in both rural and urban communities of the West Coast region, because (i) much of the research on health seeking in The Gambia focuses on rural communities [17–19]; (ii) and few studies explicitly consider the differences between women living in rural and urban areas, seemingly assuming the homogeneity of women’s lives and experiences concerning infertility [20].

This research is guided by the following research questions: (i) what are the health seeking paths of women with infertility living in the West Coast region of The Gambia? (ii) What is the influence of aetiological beliefs on their health seeking paths? This research topic is important since infertility strongly affects the lives of many women in The Gambia. The study is particularly relevant because of the limited research in this area for almost twenty years [2,16] and due to the changing political situation, with resulting opening up of opportunities in the health system [21]. The former president of The Gambia Yahya Jammeh (in power between 1994–2017) portrayed himself as having powers to cure infertility, claiming that Western tight jeans and underwear made the women of his country ‘barren’ [22,23]. The current First Lady Fatoumatta Ba Barrow is involved in a campaign to raise awareness of the issue of infertility [24]. Additionally, research on other health domains has shown much change in health seeking behaviour in The Gambia over the past decades [25], highlighting the importance of understanding these trends and dynamics in relation to health seeking among women with infertility. This will also help generate demand among, and improve the responsiveness of the health system to, those most in need.

**Fig 1:**
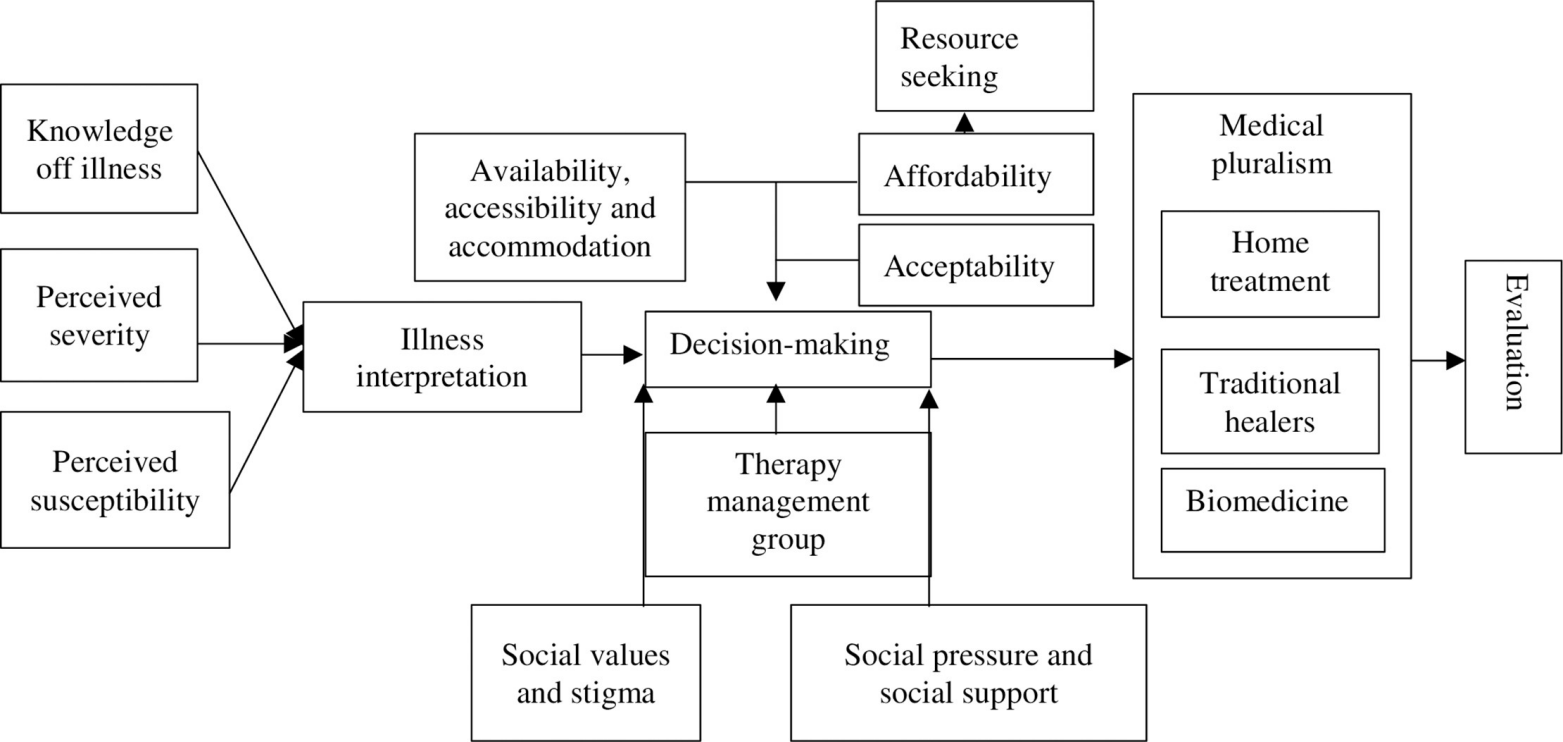
The PASS-model for health-seeking behaviour and access to care research [26].

Considering this complex background, a comprehensive framework is needed to understand the health seeking behaviour of women with infertility living in the rural and urban areas of the West Coast region of The Gambia. The Partners for Applied Social Sciences (PASS) model for health seeking behaviour and access to care research [26] (Fig 1) is a theory-based model that consists of five main categories (i) illness perception and explanatory models (i.e. perceived severity and susceptibility, knowledge of illness and illness interpretation); (ii) access to treatment and resource seeking (i.e. availability, accessibility, accommodation, affordability, acceptability and resource seeking); (iii) social values (i.e. social values and stigma, social pressure and support, and therapy management group); (iv) medical pluralism; and (v) evaluation. The PASS-model is based on the merging of different access and health seeking behaviour models to provide an easy-to-use tool. This model was first developed to understand health seeking for malaria but is adaptable to different contexts and research questions related to health seeking behaviour more generally [17,27–30]. It guided the current research by providing a conceptual background, informing the data collection by allowing us to develop more comprehensive interview guides and providing a starting point for the grounded thematic analysis of the data. To the authors’ knowledge this is the first study on health seeking of women with infertility informed by the PASS health seeking and access to care model, worldwide.

The pathway model illuminates how different factors influence people’s health seeking path and was therefore deemed most appropriate for exploring and understanding the health-seeking behaviour of women with infertility [26]. The analysis of the data is presented according to the five sections of the PASS model. The result show how Gambian women living in rural and urban communities made use of biomedical treatment as well as indigenous treatment provided by local healers, sacred places and *kanyaleng* groups. While aetiological beliefs inform women’s health seeking path, other more structural factors such as the affordability of biomedical health care, the limited availability of biomedical treatment and respondents’ family and social network are at least as important. The perceived effectiveness of the treatment available and the duration of the fertility problems influenced women’s pragmatic decision to visit different health care providers resulting in a complex and dynamic health seeking path. These results show the limitations of attributing treatment delay, the choice for non-biomedical health care and limited adherence solely to ‘traditional’ beliefs.

## Methods

### Research design

This research is part of a wider anthropological and health systems research project designed to understand the lived experiences and access to care of men and women with infertility in Senegal (Casamance) and The Gambia (West Coast region). Fieldwork was conducted for a period of four months between September 2017 and May 2018. Qualitative methods were triangulated to ensure the in-depth nature of the research, and to enable the researchers to reflect on local narratives and personal stories of the participants. During the period of fieldwork, secondary data sources were collected for analysis to sustain the results of the qualitative data collection and provide a more contextualised understanding of the research question. When respondents were fluent in English, data collection took place in English by the first author. However, in most cases informal conversations, interviews and group discussions were carried out with the assistance of a local trained translator (BJ) (with comprehensive previous experience in qualitative research) in Mandinka. The translator was trained extensively for four days and familiarized with the themes. The translator was supplied with the question guides so that the first author and the translator could discuss the terminology, phrasing and translation of potential questions. During data collection, the translator and the first author worked together as a team and translation occurred simultaneously. Attention was paid to non-verbal communication such as body language, gestures and silences. The local concepts used in the paper are in Mandinka, since this was the main language spoken by the respondents.

### Study site and population

This qualitative study took place in rural and urban communities in the West Coast region of The Gambia. In The Gambia, the Mandinka are the largest ethno-linguistic group comprising 42% of the population, followed by the Fula (18%), the Wolof (16%), the Jola (10%) and the Serahule (9%) [25,31]. Islam is the pre-dominant religion (90%), 8% are Christian and 2% other. Research on infertility rates in The Gambia are quite old, with a contraceptive prevalence study conducted in 1993 stating that around 2% of women at the end of their reproductive period had no children and considerably more women (11.6–15.2%) had a longer than expected time interval since their last birth [32]. Another study found 9.8% of participants to be infertile, with secondary infertility (defined as no pregnancy after at least 12 months of regular unprotected sexual intercourse) affecting 8.8% of all participants [33].

The rural study site was an hour’s drive from the urban area and chosen because of the cultural proximity of the fieldworker and the geographical location at the outskirts of the West Coast region. Following the ethnic distribution within the village, most respondents were Mandinka, Jola and Karoninka and respondents were predominantly Muslim. Many respondents worked as gardeners during the dry seasons and as rice farmers during the rainy season.

The urban women with infertility interviewed for this study came from various urbanized communities such as Bakau, Latrikunda, Lamin and Tallinding. As a result of migration from rural areas to this urbanized area, the population and our correspondent study sample is very diverse in terms of ethnicity, education level, class and corresponding life styles. As in the rural study site and conforming to the overall distribution of the population, most respondents were Mandinka and Muslim. While some of the urban respondents were illiterate and lived from subsistence farming and informal activities such as selling vegetables in the local market, others were highly educated with a secure job in the public sector or owning a private business.

### Positionality of researcher

Prior to carrying out this research, the first author had resided in The Gambia (in total 14 months over the course of three years). She was informally adopted by a local family and acquainted with culturally appropriate expressions and behaviour. Her position as a married woman was important in facilitating discussion around infertility, at the same time her position as a white, Western woman made her at times an outsider. This twofold position as insider and outsider was helpful for some of the respondents, as it made the researcher a confidential conversation partner for those who wanted to share their stories and secrets.

### Data collection tools

#### Participant observation and informal conversations

In total 18 observations and 42 informal conversations were formally written down during or immediately after the observation and conversation occurred. The researcher was always open about her identity as researcher during observations and informal conversations. Participant observation happened throughout the period in the field and incorporates participating in everyday activities and carrying out informal conversations with a maximum variety of research subjects both in the urban and rural communities. Multiple visits were carried out to public and private health centres (at the following days: 5/10/2017; 27/11/2017; 28/11/2017/; 06/12/2018; 06/02/2018; 12/02/2018; 06/04/2018). Also, indigenous healers were consulted (31/01/2018; 02/02/2018; 12/02/2018; 16/03/2018; 18/03/2018), trips were made to the sacred places of Folonko (Kartong) (19/01/2018–21/01/2018) and Katchikaly (Bakau) (1/12/2017; 10/04/2018) and performances of *kanyalengs* observed (12/10/2017; 14/10/2018). These methods allowed for a more contextualized understanding of complex research questions with regard to biomedical and local conceptions of health and women’s health seeking behaviour. These methods also facilitated gaining access to respondents and building trust between the researcher and participants. This was particularly important given the sensitive nature of the research topic and questions, allowing to minimize socially desirable or otherwise biased answers.

#### Interviews

In total 36 semi-structured interviews were conducted with women with infertility, of which 21 interviews with women living in the urban area and 15 with women in the rural area. More interviews were carried out with women living in the urban area because of the focus of the broader research project. When appropriate, respondents were interviewed multiple times to aid the in-depth understanding of specific issues. Most interviews were recorded; however, if the interviewee preferred not to be recorded, the responses were written down in detail during or directly after the interview. Interviews were conducted at places were respondent felt at ease such as the privacy of their residences, the houses of trusted friends or an empty room of local organizations working with women with infertility. A first question guide was developed based on the PASS-model, but further refined based on emerging results (S1 File). The guide was used to structure the interviews but allowed for flexibility.

#### Group discussions

In total, 7 group discussions were conducted, transcribed and when necessary translated into English. Four group discussions were conducted among women with infertility, three group discussions consisted of a mix of women with and without infertility problems. Group discussions were used to gather different views on a particular topic and made use of the same question guide (S1 File). Group discussions took place in several places including the privacy of people’s homes and public spaces such as restaurants.

#### Free listing and pile sorting

Four of the interviews and one group discussion with women who were infertile made use of the methods of free listing and pile sorting in order to unravel the aetiology of infertility. In the free listing method, women were asked to cite all the causes of infertility. Subsequently, these were noted down on separate cards which women had to sort into coherent piles. This method demonstrated the complexity of local aetiological classifications and proved to be useful for relating aetiology to health seeking behaviour.

#### Secondary data

The study reviewed media outputs [34–87] before and during the period in the field and reports and pamphlets regarding topics on infertility and gender published by the Gambian government and non-governmental organizations present and operating in the country [88–95]. The analysed secondary data were used to inform and cross-check the analysis of the primary data collected. These documents were useful to understand social norms, the health system in general, the availability of previous and current infertility treatments and provided a socio-political framework within which to further understand the topic.

### Sampling

During the fieldwork, participant recruitment was continuously carried out through a mixed-sampling strategy. There were no *a priori* inclusion criteria, except for being an adult, willingness to provide informed consent and perceiving oneself as having fertility problems. Purposive sampling was utilized to enable maximum variation of identified participants through a gradual selection process. Maximum variation in the selection of respondents ensured the representativeness of different groups in the study sample. The research participants were (i) identified during participant observations and informal conversations at community level; (ii) with the assistance of local organizations; and (iii) through snowball sampling, whereby respondents identified other potential respondents.

### Data analysis

Qualitative data collection and analysis were performed concurrently and data analysis was an iterative process. Preliminary raw qualitative data and secondary data were intermittently analysed, and preliminary results were then translated into the question guides for follow-up data collection. Initial results were continuously confirmed or refuted in the field, until theoretical saturation was reached. When returning from the field, interviews and group discussion were further transcribed. When data collection occurred in Mandinka, the translator was instructed to directly translate the responses of the respondents in Mandinka interviews into English. The translation of local concepts was discussed and double-checked for accuracy. The resulting texts were thematically analysed. Data collection and analyses were guided–but not determined—by the PASS-model for health seeking behaviour and access to care (Fig 1) [26]. This indicates that the researcher applied principles of grounded theory: an initial coding framework was established based on the results, which was further deepened by looking into the existing literature. The PASS-model provided an analytical framework to compare and contrast the data. In a last step, the data were analysed in order to understand which social positions (e.g. location, age, socio-economic status, ethnicity, religion) were relevant for women’s experiences. The systematization and analysis of all qualitative data was carried with NVivo 11 Analysis Software (QSR International Pty Ltd. Cardigan UK).

### Ethics

The study was approved by The Gambia Government/MRC joint Ethics Committee (SCC 1562) and by the ethical commission of Vrije Universiteit Brussel (Belgium). The interviewers followed the Code of Ethics of the American Anthropological Association (AAA). This implied that all interviewees were informed about the study, the topic and type of questions as well as their right to decline participation or to interrupt the conversation at any time before the interview. Written informed consent was obtained before each interview, which was documented either with a signature or with a thumb print. When consent was not obtained, people were automatically excluded from the study sample. Anonymity was guaranteed, and confidentiality of interviewees assured by using only descriptive identifiers and assigning a unique code to each data source.

## Results

To comprehensively delineate the complexity of the data, we present the findings of the thematic analysis of the data according to the PASS model in five sections: (i) illness perception and explanatory models; (ii) medical pluralism; (iii) social values; (iv) access to treatment and resource seeking and (v) evaluation (Fig 1) [26].

### Illness perception and explanatory models

#### Perceived susceptibility and severity

Overall, women from rural and urban areas perceived themselves to have fertility problems when they failed to become pregnant after one to two years of having unprotected sex with their partner, or when they had a miscarriage during this period. In a context of high levels of migrating men, some women were living without their husbands for several years. If they did not become pregnant during the short period (3–6 months) when their husbands were present, they also began to consider themselves to be infertile. Interview and informal conversations showed that in most cases women were blamed and stigmatized by community and compound members and sometimes also by their own husbands. Regardless of diagnosis, women were far more likely to seek health care compared to men.

While some women expressed that infertility could also be caused by their husbands, or that they were spending too much money searching for treatment that was unlikely to be helpful, they found it difficult to talk with their husbands and to encourage them to go for testing, diagnosis and treatment. Our data imply that mainly (i) men who were already married before without having a child and (ii) younger (25–35 year) and educated men who were married with a woman of their own choosing (as opposed to arranged/forced marriages) were more likely to seek diagnosis and health care. That it however remained very sensitive to involve the husband in the health seeking becomes clear from this quote by an adult urban woman with infertility: *The doctor told me*: *“We have done so many hormones tests*. *You have been here every week for ultrasounds and you have been ovulating*. *So*, *we want you to bring your husband*.*” I said*: *“No because he is okay*. *He already has a son*. *I told you that he is okay*.*” The doctor told me*: *“do you know how many women I treated here*, *they would come with their pregnancy and deliver and we eventually found out that the husbands are not the fathers of the baby*?*”*.

#### Knowledge of illness and illness interpretation

Overall, women said there was limited information available on the prevention and causes of infertility. The data showed that the general aetiological understanding of infertility was remarkably similar in the rural and urban area: the causative factors were categorized by the women as either supernatural (i.e. factors originating from outside the body that are undetectable through biomedical approaches) or natural (i.e. factors originating within the body, detectable through biomedical approaches). Though women acknowledge that there might be multiple causes at the same time, the analysis of the specific illness narratives showed that women commonly allocated their fertility problems to one specific origin which could be either supernatural or natural. While there is a broad range of potential causes of infertility, our data show that urban and rural women allocated their problems mainly to *kuntofengo* (supernatural aetiology), *seketoo* (natural factor–folk illness) and fibroids (natural factor).

#### Supernatural aetiology

*Kuntofengo* was the most frequently mentioned reason for women’s inability to become pregnant or for miscarriages. The origin is attributed to *jinnoo* (i.e. invisible spritis) who tend to infect beautiful, talented and smart women from birth onwards. Respondents mentioned symptoms such as headaches, erotic dreams or nightmares in which symbolic elements such as animals or *kankurangs* (a family of masquerades wearing a costume made from tree bark fibres and leaves; they are commonly associated with the spiritual world and have superhuman powers) are present. As an elderly urban woman with infertility explained:

*If you have kuntofengo and you missed your period*, *it comes as a dream*, *like you will dream of a man who is having intercourse with you*. *When you wake up in the morning you see that you had a miscarriage*.

Few women also attributed their infertility to witches *(buwaa)*. These are evil men and women with a power called *kumfanuntewo* (literally translated as ‘wide-headed’). People with this power are perceived to have the ability to see and interact with the spiritual world. In exchange for fortune, witches catch human souls, drink their victims’ blood and eat human flesh. Respondents explained that witches can change into different forms such as birds and snakes. Pregnant women are advised to sleep inside during the night in order to avoid a witch in the shape of a bird putting a bad spell on them. It was explained that pregnant women could also be witches, and since they had eaten other people’s children, they were part of the credit and debt system of the witches’ club and had to pay the highest price (i.e. their own children). Therefore, sometimes community members accuse women with infertility of being witches and eating their own children in the womb.

*Some will accuse the woman of being a witch and drink up her children in her womb*. (Adult urban woman with infertility)

Some of the interviewed women with infertility perceived themselves to be victims of ‘black magic’ coming from a marabout (mostly male healers affiliated with Islam). The principal domain of marabouts is the protection and treatment of afflictions caused by *buwaa* and *jinnoo*, but they are also seen to be capable of causing infertility. While everyone can ask marabouts to give someone infertility, co-wives and family members–especially the mother-in-law–were commonly accused to bring such big misfortune.

#### Natural aetiology

In the discussion of the PASS-model, particular attention is given to the notion of folk illnesses, i.e. syndromes for which a culture provides an alternative aetiology, diagnosis and healing measures [26]. In terms of natural factors causing infertility, an analytical distinction can be made between folk illnesses (*seketoo* and *buluntoo*) and other natural causes which largely follow biomedical notions of infertility.

*Seketoo can cause someone not to have a child*, *because when you have seketoo it drains your body until you become very tiny and dry*. *Even when you become pregnant it spoils and becomes menstrual blood*. (Elderly rural woman with infertility)

The symptoms of *seketoo* are (i) itching in the genital area which can lead to bleeding, (ii) white coloured discharge and (iii) a small bumpy structure inside the vagina. To explain this bumpy structure of *seketoo* the respondents made references to similar structures in animals. For example, respondents explained that a fish has a small round structure in its throat that is similar to bumps associated with *seketoo*. Women who had *seketoo*, often did not know how they got infected by it. Some people thought it just grew naturally, while others explained you get it by stepping over fish scales on the ground. Younger educated women explained that the concept of *seketoo* is often used for any problem in the vaginal area including urinary tract infections (UTIs) and sexual transmitted infections (STIs). While *seketoo* is a well-known disease, less information is available about *buluntoo*. *Buluntoo* was characterized by abdominal and back pain. It was described as a stone in the womb causing fertility problems. Various causes were given: eating too much pepper or lime, sitting bent over a food bowl, walking on water used for laundry, using the same bathroom and walking on herbs placed by people on traffic junctions.

Many urban respondents explained their fertility problems were caused by fibroids. Fibroids were understood to be an obstacle inside the womb of unspecified origin. Other aspects which could lead to infertility were abortions (*konobondoo*, literally translated as stomach removing), ‘overdoses’ of family planning tablets and injections.

*I heard from people that my brother’s wife used to take the family planning tablets before she got married*. *Now that she is married she could not become pregnant and people said that she overdosed herself with the tablets*. (Elderly urban woman with infertility)

Many respondents said that stress was an important reason for women’s inability to become pregnant. Some people said that their miscarriages were caused by the hard work they needed to do in the compound, farms and gardens. Few women with infertility knew that secondary infertility could be caused by infections after delivery. Other perceived natural causes are a *tight* vagina and small hips making people unable to become pregnant or to give birth; having a *weak womb* so that the sperm runs out after intercourse or leading to miscarriages; having *hot water* inside the stomach leading to miscarriage. Some respondents mentioned that when the blood of husband and wife did not match, this led to infertility.

### Medical pluralism

Observation, informal conversations, interviews and group discussions indicated that various options are available for women with infertility in the West Coast region. Following the PASS-model, a distinction is made between home treatment, traditional/indigenous treatment and biomedicine (Fig 1) [26]. Respondents’ opinions about the compatibility of combining treatments varied; some believed that indigenous and biomedical treatment options were not compatible, while others would easily combine several treatment options at the same time. In this study, only one woman had never been to an indigenous healer for infertility treatment. She had studied abroad and came from a family with a high socio-economic status.

#### Home treatment

Home treatment consisted of consuming locally known parts of trees, herbs or leaves to alleviate symptoms and as remedies. These trees, herbs and leaves are found in the direct surroundings. A well-known remedy is called *juttoo;* it is made of roots that *clean the stomach* to restore the womb after miscarriages. Mainly people living in the urban area occasionally purchased contraception in order to let their womb *rest* after multiple miscarriages. As an adult urban woman with infertility explained:

I took the injection for three months after my miscarriages but after the three months I did not support it because I did not menstruate during that time and the second thing was I always encounter abdominal pain.

#### Indigenous healing

*Marabouts*. When people thought their infertility was caused by supernatural powers, treatment was beyond the scope of biomedicine and the only healers that can help were marabouts. The concept marabout is used for a wide range of healers who cure based on Islamic verses to give their medicine power. It is also sometimes used for herbalists who rely to a lesser extent on Islamic verses. Observations showed that these indigenous healers have numerous ways of diagnosing their patients, including communication with *jinnoo* and analysing the patterns of cowrie shells, stones or lines in sand. Respondents said that during consultation they received treatment based on the symptoms they explained. Various treatment options were available, commonly it consisted out of *safoo’s*. These are small papers on which verses of the Quran are written, and then folded into a small bundle and wrapped in leather. These amulets are worn on the body, for example around the head when it concerns headaches caused by *kuntofengo*. Another treatment is *nasoo’s* which is made by writing down words or verses of the Quran on paper or a wooden slate and then put into water used for bathing and drinking.

*Somebody told me about one marabou at village X*. *When I went there he gave me holy water and some medicine but he also told me*: *“be very careful because there is one person in your compound who didn’t want you to marry you husband”*. (Adult rural woman with infertility)

Some respondents and media reports discussed the treatment provided by the previous president, Yahya Jammeh, who considered himself a powerful marabout. He prescribed and made treatment for women facing reproductive challenges.

*Before Jammeh left*, *nobody dared to talk about infertility*. *Nobody would say “you need to go to the hospital” because he had his own program […] Some women claimed to be cured by him*, *however*, *they did not have many other options than to say this*. *You could not encourage people to go for a scan in the health centre*. (Adult urban woman with infertility)

*Herbalists*. Herbalists work solely with herbs. They are able to cure natural aetiological factors associated with infertility. In case of *buluntoo*, all respondents agreed that indigenous healers from the ethnolinguistic group Jola knew the best treatment. Our data indicated that medication often contained bark, leaves or roots, which are put in water and have to be swallowed on a regular basis. The resulting diarrhoea was perceived to *wash the stomach*. Many of the remedies against *seketoo* have to be vaginally applied, a male herbalist showed us his medication consisting of a cream with unspecified ingredients which had to be inserted vaginally with some cotton. An adult rural woman with infertility explained to us a cure for *seketoo*:

*There is Mandinka medicine called ‘simanko la’ meaning ‘after dinner’, while others called it ‘nbenki’ meaning ‘aunty’. It has two different names but it is the same medicine. When you want to buy it, that’s the name you use, but the real name is tobacco powder. People say it used to cure seketoo, but I am not very sure because when you apply it, you end up crawling on the ground, that medicine is not a joke […] It cause dizziness, and you will not be able to talk and it makes you vomit all the time […] some encounter diarrhoea*.

*Kanyaleng kafoolu* (plural, singular *kafoo*). These organizations consist of women with fertility problems or who have experienced child loss. Respondents explained that membership serves both as a coping mechanism and as a proactive effort to overcome infertility, subfertility or child mortality. Observations and interviews show that through becoming a *kanyaleng* these women want to *beg to God* to heal them and perform miracles. While initiation rituals differ and change throughout time and place, a common idea is that during initiation the evil spiritual forces are driven away, e.g. by beating the initiated or having a *kankurang* present.

*Fortune-tellers*. Some women with infertility also visited fortune-tellers commonly after several years of looking for treatment. A common advice these fortune-tellers give is to move to another village where these women would be able to become pregnant and deliver. There are multiple possible reasons to move: a good healer could be present in the new village or the *jinnoo* could not follow these women. The time people spend away can be quite long, as a woman resided over three years outside her village to receive medication by a marabout.

*Female circumcisers*. Secondary data documents that female circumcision recently became illegal in the country, but all respondents still knew elderly women who used to perform the circumcision. In case of *seketoo* female circumcisers are also consulted. Various women reported how *seketoo* got cured by cutting away the bump inside their vagina. This adult urban woman with infertility described her experiences:

*People use to say that seketoo can stop somebody from having a child but I have done the treatment for seketoo. The day they removed the seketoo, that day I bled a lot until I fainted. Even the doctor that helped to stop the bleeding, told me to sit on an ice block, because I was bleeding severely when they cut the seketoo […] The old woman that did it to me, used to circumcise people*.

*Sacred places*. In addition to these indigenous healing methods, women with infertility from rural and urban areas visited sacred places which hold a special significance for women with infertility. The most well-known places are the crocodile pools of Katchikaly in Bakau and Folonko in Kartong. Observations and interviews show that women with infertility go there to pray, drink or wash themselves with the holy water of the pool. Many rituals performed by herbalists, marabouts and *kanyaleng kafoolu* from the area were performed at these sacred places. Observations taught us that when entering these sacred places, a donation consisting of salt, sugar, kola nuts and white candles was given to the old ladies safeguarding the pools. After the purpose of their visits was explained, the women sat in front of the pool to pray and to conclude the whole group of visitors and safe guarders prayed together. Visitors took some of the sacred water from the pool with them and some showered with water from the pool.

#### The biomedical sector

When the perceived aetiology is allocated to natural factors people will visit the biomedical sector consisting of private and public health centres. The health seeking itineraries would commonly bring rural women first to local health centres, while urban women often went directly to The Edward Francis Small Teaching Hospital (i.e. the main tertiary level referral hospital in the capital city Banjul). An adult woman told us a story about (somebody claiming to be) a nurse wanting to treat her *seketoo* by cutting away her clitoris:

*There was a man who fooled me. Still now I won’t forget. He said he is a medical nurse, he said I have an UTI […] That is seketoo. In our tradition we have the believe that it can be cut. Now I know that I do not have it, but at first I thought it was true and that I had it. He wanted to have money from my husband, and the nurse said the solution is to cut it out, and my husband asked him the price, he said D1000 [21 USD] [laughing] […] My husband gave the nurse the money […] When I went to his place, on the whole he did not know what is an UTI. So, he was checking me and touched my clitoris and I told him “that’s not the one”. Then I told him: “you have taken the money just go your way”*.

In comparison with the public health sector, the private health centres were perceived to be of better quality due to better equipment, treatment of patients and the higher level of motivation of the health staff. Highly educated and young women explained that they preferred to go for ‘Western’ medication first because it was based on diagnosis and specified measurements of drugs.

### Social values

#### Social values and stigma

Women with infertility were stigmatized in their communities due to their conditions. This encouraged them to look for health care. In the country, men and women find it difficult to discuss sexuality and pregnancy and some women found it difficult to report their problems with the doctor and mentioned vague complaints such as stomach-ache or menstrual pain when presenting. Educated women often had less difficulties to discuss their problems with health staff and knew about biomedical causes of infertility. Stigma was not a major barrier for women visiting biomedical health care providers, as they were primary concerned with overcoming their condition.

#### Social pressure and social support

While there was a lot of social pressure by the broader community, the family and the husband to have children, interviews and observations illustrated that health seeking was for most women in the beginning an individual and private path. Despite the desired secrecy in the initial stages, the social networks of the respondents are important resources. Rural women explained that although they resided further away from gynaecologists, the strong social network in their village was very helpful. The *kanyaleng kafoo* in the rural community within which this study is situated provides social, emotional and sometimes even financial support to women with infertility. Given that there is no clear information on where to go for infertility treatment, people living in urban and rural areas relied mostly on verbal recommendations from friends, family and referrals by health providers. A young woman with infertility living in the rural area started discussing her problems with the people close to her after three years in her marriage:

*I do not keep my miscarriages secret. I discuss it with my mother and people who experience the same thing as I do. My mother is really worried about my childlessness and when she hears of any place [that offers treatment], she informs me*.

#### Therapy management group

Some women were able to discuss their fears and concerns with their husbands, others discussed it with few close family members, friends and occasionally employers. These people could be part of the therapy management group, the people who are involved in health seeking with, or on behalf, of the sufferer (Fig 1) [26]. While the therapy management group mainly consist of women with the exception of the husband, in the case of one young urban woman also the brother-in-law helped. In the urban area some young women received financial assistance from employers to look for biomedical health care.

### Access to biomedical treatment and resource seeking

During group discussions and interviews, it became clear that rural and urban women with infertility were keen to attend the biomedical health care sector to find treatment. Older women also said they wanted their daughters with infertility to have the opportunity to go to the biomedical health care sector, in addition to with what they described as *the more traditional ways of healing infertility*. In what follows, the barriers in access to biomedical health care are described in relation towards women’s social position. Though the PASS-model also mentions the factors accommodation and acceptability (Fig 1) [26], these factors were not found to be relevant in this study. Even participants living in rural areas could drive to the urban area and back within a day, and many also had family members living in the urban area, where they could reside in case of emergency. In terms of acceptability, all women accepted biomedical health care.

#### Availability

Interviews and observations taught us that many of the gynaecologists work in both the public and the private sector. Private clinics are mostly located in Banjul and the surrounding urbanised area. Secondary data analysis showed that fertility treatment, if available, is limited in The Gambia: conventional methods of infertility treatment involve the surgical repair of blocked fallopian tubes and blind hormonal stimulation with Clomid. Tests that can be done are routine blood- and urine analysis, a blood test to screen hormones and sexually transmitted diseases, semen analysis, an ultrasound scan of the womb and ovaries and a contrast X ray of the womb to determine whether the fallopian tubes are open.

#### Affordability

Women facing reproductive challenges spend a significant part of their savings to get pregnant. Both in rural and urban communities, the kind of infertility treatment women used depended on what they could afford. In the West Coast region of The Gambia, there were strong inequalities in terms of financial resources. Except for the few respondents who were financially independent or had insurance, the costs associated with the private health sector put this out of reach for most. For many respondents the costs associated with presenting to the public health sector were also too high because some of the laboratory tests had to be conducted and/or drugs purchased outside the health centre.

*Now in rural community X*, *it is not difficult to know where the best hospitals are or to know where certain services are done because people have a cell phone*. *They can call to people living in the urban areas*. *So*, *it is easy to get to know*. *People’s beliefs in traditional things is the same in the rural as in the urban area*. *Transport is not a big difference in terms of affordability*. *If you can pay for the health centre and the medication*, *you can also pay for the transport*. *Money is the only difficulty*. (Adult rural woman)

#### Accessibility

During the first years of health seeking decisions were made based on convenience of travelling. With no gynaecologists readily available in the rural area, women living in the rural area need to travel. For some women in the rural areas, even the 30 Dalasi fare (approx. 0.6 USD) to travel to the urban communities West Coast region for biomedical health treatment is an important hurdle. However, women who could not afford the small transport costs could also not afford to visit the biomedical health facilities. Accessibility is also complicated because the private and public health sector often require multiple visits for a diagnosis and possible treatment. This is difficult for people with a low socio-economic background living in the rural area, for example a young woman living in the rural community told me her husband went to the urban area for semen analysis but by the time he arrived the sperm was spoiled. Only women who were economically independent could travel abroad to find more advanced biomedical diagnostic and treatment services.

#### Resource seeking

Respondents waited extensive periods of time between visits to the health centre trying to save enough money for the next steps or contacting people within their social network who could help them financially. There were few reports of husbands inhibiting wives from visiting the biomedical health sector due to the financial burden imposed. The social pressure to become pregnant is often so strong that even women with limited financial resources look for the necessary funds to access the public health sector at least once.

### Evaluation

Respondents did not always recognize symptoms because these were either absent, diffuse or difficult to interpret. Facing uncertainty about their body and the aetiology, many women interviewed follow a trial and error search for relief and meaning.

*I went to biomedical doctor A*, *indigenous healer B*. *I also went to Banjul to the surgeon*, *he is the one who washed my stomach but I still had many miscarriages*. *I also went to my own village to seek treatments*. *I also went to Casamance [South Senegal] a place called C*. *I also went to indigenous healer D in village E*. *I went to village F for the same purpose*. *I also went to the marabout in G*, *he told me that there is no sickness with you and no jinnoo and you will have a child*. *He also told me*: *“you have visited many places”*, *I told him “yes”*. *He told me*: *“go and relax*: *you will have a child”*. *There is another man from village H*, *I also went to him*, *he told me that your prayers would be answered through women; and I asked more information*. *He said*: *“well I do not know but your prayers would be answered through them”*. *Then I sat for three to four months and after that the kanyalengs bathed me*. (Adult urban woman with infertility)

The process of health seeking could take up to 20–25 years and during this time beliefs about the aetiology often changed. An urban woman from a low socio-economic background visited indigenous healers for ten years and even travelled to Guinea Bissau [West African country] to visit a marabout. Given that her abdominal pain did not reduce and she remained childless after ten years, she went to the biomedical health service where she received the following information:

*They told me that the fibroid is very big and the instrument to be used for the operation so that it will not affect my womb is not available in this country, unless I travel aboard. So, I even travelled to Senegal and the price they charged me for the operation there, I couldn’t afford it. I came back to The Gambia and I agree for them to remove it together with the womb*.

Despite women’s initially held beliefs and knowledge about the causes, the perceived aetiology often altered in accordance to the prolonged nature of their condition, the lack of effectiveness and sometimes the side effects.

*Many times*, *I stopped a treatment*. *If I used one treatment for a long time and I didn’t see any benefit I stopped and moved to another place*. (Adult urban woman with infertility)

## Discussion

This qualitative study aimed to understand the health seeking behaviour of women with infertility and the influence of aetiological beliefs on health seeking paths. To the knowledge of the authors, this is the first study exploring the health seeking of women with infertility using the PASS-model as basis for qualitative enquire (Fig 1) [26]. This model allowed for a detailed and systematic analysis of the different aspects of health seeking behaviour relevant to women living in the West Coast region of The Gambia. This study showed that health seeking behaviour is strongly influenced by the limited availability and affordability of biomedical diagnosis and treatment, people’s social network and to a lesser extent by varying illness perceptions. In addition, this study contributes to the few earlier qualitative studies looking at the influence of social positions on the lives of women with infertility in low-and middle-income countries [96,97].

Insights into the complex beliefs systems with relation to infertility show that the interpretation of the causes and symptoms of infertility were quite similar between rural and urban women in the West Coast region with most women attributing their fertility problems to *kuntofengo*, *seketoo* and fibroids. When it comes to people’s understanding of folk illnesses (*buluntoo* and s*eketoo*) and fibroids, more research is needed. It could be helpful for the prevention of infertility if public health messages link local disease classifications (*seketoo* and *buluntoo*) to UTIs and STIs [19]. While educated women would perceive these as UTIs and STIs, it is interesting that even some of these women perceived that cutting *seketoo* is the best treatment. Currently, folk illnesses are often ignored in public health practice and this might imply delays in accessing biomedical health care. Mainly women in the urban area perceived fibroids as an important cause of infertility, though they had limited knowledge about the causality and treatment. In other SSA countries similar lack of clarity about fibroids have been found, for example in Gabon the folk illness *zchaw* (Fang) has symptoms similar to fibroids [98], and a study among women with uterine fibroids in Lagos (Nigeria) found that these women had limited knowledge about the aetiology and treatment of fibroids [99].

In many cases, there were no specific symptoms of infertility and most women go through a serial process of trial and error that is guided by the perceived improvements of the women’s condition, the prolonged nature of infertility problems and sometimes the perceived side effects of treatment. These sometimes extremely long and complex itineraries illustrate that urban and rural women are actively seeking to understand their problems, the underlying causes of their misfortune and the healing process. People’s social networks proved to be important for their health seeking behaviour. An important added value of the PASS-model is that it recognizes that people are part of households, families and communities, which is in contrast with several other models that are based solely on individuals [26]. The therapy management group proved to be relevant for women’s decision making as it was an important source of information, financial and social support.

Most urban and rural women consulted various health sectors, including remedies at home, indigenous treatments and the biomedical public sector. Commonly women with infertility did not prefer one type of treatment over another, indicated by the frequent alternation and occasional combinations of treatments for infertility. All women wanted better access to good quality biomedical health care for infertility. Educated women with infertility did have an outspoken preference to attend private health facilities for diagnosis and treatment, but for many of them this was not possible due to financial reasons. This study found that affordability of diagnosis and treatment dictated the pragmatic decisions of women with infertility in the West Coast region when it comes to looking for health care. The associated costs with going to the public and private health centres proves to be a major barrier to attend the biomedical sector and makes it difficult to adhere to the multiple visits required.

Availability was also an important barrier to access biomedical health care. At the national political level, the management of infertility appears not to be a priority for the health sector (though this may change in years to come, especially with involvement of the First Lady). This may be partly explained by claims the former President made regarding his ability to cure infertility as well as a great many other competing demands [23,41]. His statements and approach put limits on the political space to develop national health policies and attract international funding related to these issues. This is particularly sad, as the high rates of infertility in SSA are partially explained by reproductive tract infections, including STI’s and pregnancy-related sepsis, which would in many cases be preventable with early detection and appropriate antibiotic treatment [4,14]. Even in the private health centres there are very limited current services for infertility in the biomedical public health sector in The Gambia [32]. Assisted reproductive technologies, including low-cost ones, are absent and unaffordable for most Gambian couples. This is a harsh reality, given that only a few privileged are able to travel abroad to access biomedical health facilities.

As in many other social contexts [15,100–107], we notice there is a pro-natal norm in The Gambia and gender imbalance with women being blamed for the inability to produce living children regardless of diagnosis or actual cause [108]. Women from all socio-demographic backgrounds wanted to become a mother as this is an important aspect of female identity. The inability to become a mother brings many financial, social and emotional challenges as women grapple with losing out on this vital role in society.

In contrast to the ascription of delay in literature on health seeking behaviour to ‘cultural’ or ‘traditional’ beliefs, this study stresses the importance of the structural inequalities–such as unequal gender roles and norms, socio-economic inequality and limited availability of treatment options—to understand health seeking behaviour. This has significant implications for policy planning since it shifts the burden of responsibility for addressing these constraints from the women to the broader policy-level.

The non-random selection of study participants is appropriate for labour-intensive in-depth qualitative studies, in particular when it concerns sensitive topics such as infertility [109,110]. This sampling technique is also advised for populations that are hard to find, such as women with infertility who are a relatively small group of the overall population and who are also stigmatized for this reason [108,109]. However, it should be acknowledged that the non-random selection and relatively small sample size limits the external validity of this research beyond women with infertility living in the West Coast region of The Gambia. For example, in this study physical distance to biomedical health care services did not seem to be an important barrier for most women living in the West Coast region. However, it should be noted that this might be an important barrier for people living in other regions given that the Demographic and Health survey [94] found that distance and affordability were important barriers in accessing general biomedical health care for women living in other rural regions of the country.

## Conclusion

The PASS-model presents a comprehensive framework for health planners and researchers interested in health-seeking behaviour and access to care [26]. The model places health seeking behaviour in a broader socio-political, cultural and economic context and identifies and relates a wide range of factors that might guide people’s health seeking behaviour. Guided by the PASS-model this study showed that health seeking for infertility in The Gambia cannot be understood in isolation from the social, political, and economic structures within which it is embedded, or without reference to aetiological beliefs. However, attributing delay, adherence and treatment choice solely to ‘traditional’ beliefs or a lack of knowledge does not represent how women make pragmatic choices in their search for health care given the context of structural inequalities. This analysis highlights that attention should be directed to open a public dialogue on infertility, as well as on the role of men when it comes to infertility. We call for health authorities to invest in providing information and counselling on issues related to infertility prevention and treatment, and draw up guidelines for the management of infertility at all levels of the health system. Lastly, while the authors would like to argue that couples have the reproductive right to access infertility treatment, more room should be created in the public sphere for alternative forms of social identity for both men and women.

## Supporting information

S1 File Question guide interviews and group discussions on health-seeking behaviour with women with infertility. (PDF)
