# Supplementary material for: ‘We are always desperate and will try anything to conceive’: The convoluted and dynamic process of health seeking among women with infertility in the West Coast Region of The Gambia
Source: PLoS One. 2019 Jan 31;14(1):e0211634. doi: 10.1371/journal.pone.0211634 (PMC6355002; doi:10.1371/journal.pone.0211634)
Supplement: S1 File — (PDF) [file pone.0211634.s001.pdf]

Question guide interviews and group discussions on health-seeking behaviour with women with infertility

Socio-demographic information
Ethnicity
Age
Level of education
Do you know how to read or write?
Religion
Main source of income
Where were you born/is this your home village?

We came to you because … Could you explain a little bit more about your situation (fertility problems)?
How did you feel about this? Do you consider it a problem?

Reasons for preference for children
What is considered the be the most important aspect of marriage? (children)
Why do people want to have children?
- *Probe: inheritance?*
- *Probe: maintain family lineage?*
- *Probe: help in the household?*
- *Probe: religion?*
- *Probe: source of joy, companionship? Symbols for the achievements of parents?*
- *Probe: is there a difference in funeral and other important rites between people who have children and don't have children?*

If people need to choose between many children or a continuous supply of children, what is the preference?
Is there an outspoken preference for boys or girls?
- What are the advantages of getting girls?
- What are the disadvantages of getting girls?
- What are the advantages of getting boys?
  - *Probe: financial help so more security?*
- What are the disadvantages of getting boys?

Causes of infertility problems
We would like to do an exercise about the causes of infertility (explain exercise and cards)?
Where would you go for each causal factor of infertility problems?

What do you think is the cause of your fertility problems? Why do you think this?

Which type of behaviour can cause fertility problems?

Could you mention all possible supernatural causes of female infertility?

Probe: not acting according to the norms regarding marriage?

Probe: witches?

Probe: is there a way to reverse this? Certain rituals? To who do you need to go in case of this problem?

Of the causes you just mentioned, which ones are the most important causes?

Could you mention all possible biological causes of female infertility you know?

Probe: contraceptives?

Probe: promiscuous life?

Probe: a bad mixture of the blood of men and women?

Probe: previous abortions? How does this influence current infertility?

Probe: sexual transmitted diseases? Which ones? (gonorrhoea, syphilis, chlamydia)

Probe: blocked fallopian tubes?

Probe: uterine fibroid?

Probe: FGM? How does this influence infertility?

Of the causes you just mentioned, which ones are the most important causes?

Can infertility be caused by both supernatural forces (e.g. jinnoo, buwaa) as 'natural' elements (e.g. seketoo, buluntoo, STI, fibroids, abortion) at the same time?

Have your ever heard about such cases?

Did your perception about the cause of infertility changed throughout time?

How come?

Probe: due to the inability of the treatment to cure?

Can you tell me a little bit more about buluntoo?

How do you get it?

It is communicable? How?

How does it manifest itself?

` Does it prevent conception?

Does it lead to miscarriages?

Can you tell me a little bit more about seketoo?

How do you get it?

It is communicable? How?

How does it manifest itself?

` Does it prevent conception?

Does it lead to miscarriages?

Flexibility

Could you explain us a little bit more about your search for treatment?

Where did you first go for treatment?

How did you get in touch with them?

To which other healers have you been to after?

What is according to these healers the cause of your problems?
When you started looking for treatment, how big was the chance you think that you got cured by these treatments?

Treatment

Home treatment

Which type of treatments for infertility can you take at home?
    Where can you find this treatment?
Are there any kind of medication you can directly find at the pharmacist?

Marabout

If you ever consulted a marabout,
    What were your experiences?
    How did a consultation go?
    Did he/she help you? How?
    How did you get to know the marabout?
    Why did you go to a marabout?
With which problems can a marabout help?
Can a marabout also help with seketoo, buluntoo, fibroids, STIs, …?

Health centre

Did you ever go to the governmental health centre, hospital for treatment? (why (not?))
    What are the major barriers to finding treatment at the hospital?
Did you ever go to the private health centre for treatment?
    What are the major barriers to finding treatment at the hospital?
Does Islam say anything about artificial insemination?
When you went to the Western doctors, how big did you think the chance was to get cured by these treatments?
Can you combine treatment from the marabout, from sacred places, with treatment from the health centre or the pharmacists?

Factors influencing treatment choices

How soon after your marriage did you start looking for treatment?
Why did you start looking for treatment at that point?
    Probe: comments from in-laws?
When you were looking for fertility treatment in the beginning, did you do this secretly?
    Why?
    At what time did you decided to be open about your treatment seeking?
What are the major factors influencing where you would go for treatment?
    Probe: availability, affordability, accessibility social perception, knowledge, aetiology, other factors?
    Probe: could you tell us a little bit more about this?
    Probe: do you think there is a difference in treatment seeking if you lived in a rural/urban space?

Who bears the cost for treatment seeking?

If yourself, how did you get the money to do that?

Is your husband involved in your treatment seeking in any way?

When did he start to be involved?

Is looking for treatment a strategy you use to prevent your husband from leaving you?

Did you seek the help from other people?

From how many people did you seek help?

From whom did you seek help? What did they do?

Treatment

Did you ever decide to stop a certain treatment due to the way you were treated?

Could you tell me a little bit more about this?

Kanyaleng membership

When did you become a kanyaleng (month/year)?

Did you join willingly or were you encouraged/forced to join?

Who encouraged/force you to join (family/neighbours)

What are the advantages of participating in a kanyaleng kafoo?

Where their certain barriers for joining this group?

How did you feel about joining the kanyaleng?

How many members are in your kafoo?

How often do you meet as group?

Where do you meet?

Which activities have you undertaken as a group?

(Income generating projects? Performances?)

How does your husband think about your kanyaleng membership/activities?

How does your family think about your kanyaleng membership/activities?

What did the initiation process consist of?

What were your expectations before the initiation?

Were these expectations realised? How come?

How are kanyaleng treated in your village?

Which type of prayers are used during kanyaleng performances? Would you use these same prayers also in other contexts? Why (not)?

Can kanyaleng meals start without prayers? Why not?

Do kanyalengs give advice about fertility treatments? Which kind of advice is given (e.g. pilgrimage to sacred place)

Do you feel better often participating in a kanyaleng ritual? Why do you that is the case?

Do you feel supported by the kanyaleng kafoo?

Note: this question guide was used to guide the questions but did not determine the structure of the overall interview/group discussions. Overall, we started with open questions and depending on the answers the interview/group discussion evolved. This final question guide integrates different versions of previous question guides to give an overview to the readers of which types of questions could be asked during interviews/group discussion.
